# Glycolysis reprogramming in CAFs promotes oxaliplatin resistance in pancreatic cancer through circABCC4 mediated PKM2 nuclear translocation

**DOI:** 10.1038/s41419-025-07431-4

**Published:** 2025-02-23

**Authors:** Rihua He, Chonghui Hu, Yuan Yuan, Tingting Li, Qing Tian, Tianhao Huang, Qing Lin, Shangyou Zheng, Chujie Chen, Zhiqiang Fu, Rufu Chen

**Affiliations:** 1 Department of Pancreas Center, Department of General Surgery, Guangdong Provincial People’s Hospital (Guangdong Academy of Medical Sciences), Southern Medical University, Guangzhou, 510080 Guangdong China; 2 Guangdong cardiovascular Institute, Guangdong Provincial People’s Hospital, Guangdong Academy of Medical Sciences, Guangzhou, 510080 Guangdong China; 3 School of medicine, South China University of Technology, Guangzhou, 510006 Guangdong China; 4 Department of Urology, Seventh Affiliated Hospital, Sun Yat-sen University, 628 Zhenyuan Road, Guangming District, Shenzhen, 518017 Guangdong China; 5 Department of Pancreaticobiliary Surgery, Sun Yat-sen Memorial Hospital, Sun Yat-sen University, Guangzhou, 510120 Guangdong China; 6 Key Laboratory of Malignant Tumor Gene Regulation and Target Therapy of Guangdong Higher Education Institutes, Sun Yat-sen Memorial Hospital, Sun Yat-sen University, Guangzhou, 510120 Guangdong China

**Keywords:** Cancer microenvironment, Cancer metabolism

## Abstract

Cancer-associated fibroblasts (CAFs) play a key role in oxaliplatin resistance in pancreatic ductal adenocarcinoma (PDAC). However, the potential mechanisms by which CAFs promote chemotherapy resistance have not yet been explored. In this study, we found that circABCC4 (hsa_circ_0030582) was positively correlated with poor platinum-chemotherapeutic response and a shorter progression-free survival (PFS) time in late-stage PDAC patients. CircABCC4 enhanced the ability of CAFs to induce oxaliplatin resistance in pancreatic cancer cells through glycolysis reprogramming. Mechanistically, circABCC4 enhanced the interaction between PKM2 and KPNA2 to promote PKM2 nuclear translocation in CAFs, leading to the transcription of glycolysis-related genes. The glycolytic reprogramming of CAFs promoted the secretion of IL-8, which in turn enhanced DNA damage repair in pancreatic cancer. Blocking PKM2 nuclear translocation abolished circABCC4-driven oxaliplatin resistance of pancreatic cancer in vivo. Collectively, our study reveals a circRNA-mediated glycolysis reprogramming of CAFs to induce oxaliplatin resistance and highlights circABCC4 as a potential therapeutic target.

## Introduction

Pancreatic cancer, recognized for its high malignancy and limited therapeutic options, presents a formidable global health issue with rising incidence and mortality rates [1, 2]. Chemotherapy, a cornerstone in managing locally advanced or metastatic cases, offers a means to enhance prognosis [3]. Notably, neoadjuvant chemotherapy can elevate resection rates, benefiting those with resectable or marginally resectable pancreatic cancer [4]. Nonetheless, the challenge of chemotherapy resistance persists, posing a significant hurdle in clinical management [5]. This underscores the urgent need for innovative strategies to overcome resistance and improve patient outcomes in pancreatic cancer treatment [6, 7].

Within the array of combination chemotherapy regimens for pancreatic cancer, folinic acid, 5-fluorouracil, irinotecan, and oxaliplatin (FOLFIRINOX) ranks among the most efficacious [8, 9]. A critical component of this regimen, oxaliplatin, plays a pivotal role in its effectiveness against this malignancy [10]. While targeted therapies for pancreatic cancer offer hope, olaparib stands out as an effective option, albeit primarily for patients with platinum sensitivity [11]. Exploring the mechanisms behind platinum resistance in pancreatic cancer and identifying targets to enhance platinum sensitivity could lead to significant advances in treatment. Pancreatic cancer patients exhibit a high degree of heterogeneity in their sensitivity to chemotherapy drugs, with the remodeling of the tumor microenvironment (TME) playing a crucial role [12]. Cancer-associated fibroblasts (CAFs), predominant stromal cells within the TME, are instrumental in synergistically evolving with cancer cells, markedly influencing the development of platinum resistance in pancreatic cancer [13, 14]. Our recent findings reveal that CAFs from platinum-resistant patients notably enhance oxaliplatin resistance in pancreatic cancer cells by secreting IL-8 and extracellular vesicles, facilitating a non-homologous end joining (NHEJ) dependent DNA repair mechanism [15]. Considering the diverse mechanisms through which CAFs modulate platinum resistance in pancreatic cancer cells, it becomes imperative to delve deeper into the biological behavior changes within CAFs linked to platinum resistance [16]. Exploring these dynamics is key to uncovering the complex interactions between CAFs and cancer cells in the resistant milieu, offering a pathway to identify new therapeutic targets.

Aerobic glycolysis, recognized as a cancer hallmark, not only advances tumor growth and drug resistance but also significantly contributes to the metabolic reprogramming of the TME [17–19]. A recent study revealed that inhibiting CAFs activation through disrupting Src SH3 domain-mediated glycolysis significantly reduces colorectal liver metastasis, highlighting glycolytic modulation in CAFs as a promising direction for future therapies [20]. Hu et al. unveiled that CAFs induce gemcitabine resistance in pancreatic cancer via monocarboxylate transporter-mediated aerobic glycolysis, highlighting the potential of targeting CAFs-cancer cell metabolic links to enhance chemotherapeutic outcomes [21]. Nonetheless, the impact of glycolytic modulation by CAFs on platinum resistance in pancreatic cancer is still underexplored, underscoring the need for in-depth studies.

Circular RNA (circRNA), a unique class of non-coding RNA, has garnered significant attention for its role in cancer progression, metastasis, and particularly in mediating drug resistance [22, 23]. Our previous research identified that circCUL2, highly expressed in CAFs, induces an inflammatory phenotype that accelerates pancreatic cancer progression [24]. Further studies revealed that elevated levels of circFARP1 in CAFs activate the LIF/STAT3 axis, promoting gemcitabine resistance in pancreatic cancer [25]. More recently, we discovered that CAFs contribute to oxaliplatin resistance in pancreatic cancer by delivering extracellular vesicle-packaged circBIRC6 [13]. However, the role of circRNA in mediating platinum resistance through glycolysis in pancreatic cancer remains uncertain.

In our study, we discovered that circABCC4 (hsa_circ_0030582) from CAFs is markedly upregulated in pancreatic ductal adenocarcinoma (PDAC), correlating with increased oxaliplatin resistance and poorer patient outcomes. CircABCC4 enhances the glycolytic process in CAFs, promoting the nuclear migration of PKM2, which in turn boosts DNA repair through non-homologous end joining, leading to platinum drug resistance. These insights unveil a previously unidentified mechanism, highlighting the circABCC4/PKM2 axis as a promising target for PDAC therapy, aiming to improve treatment responses by disrupting this pathway.

## Result

### Identification of a CAFs‑specific circRNA, circABCC4, that correlated with oxaliplatin resistance in PDAC

**Fig. 1:**
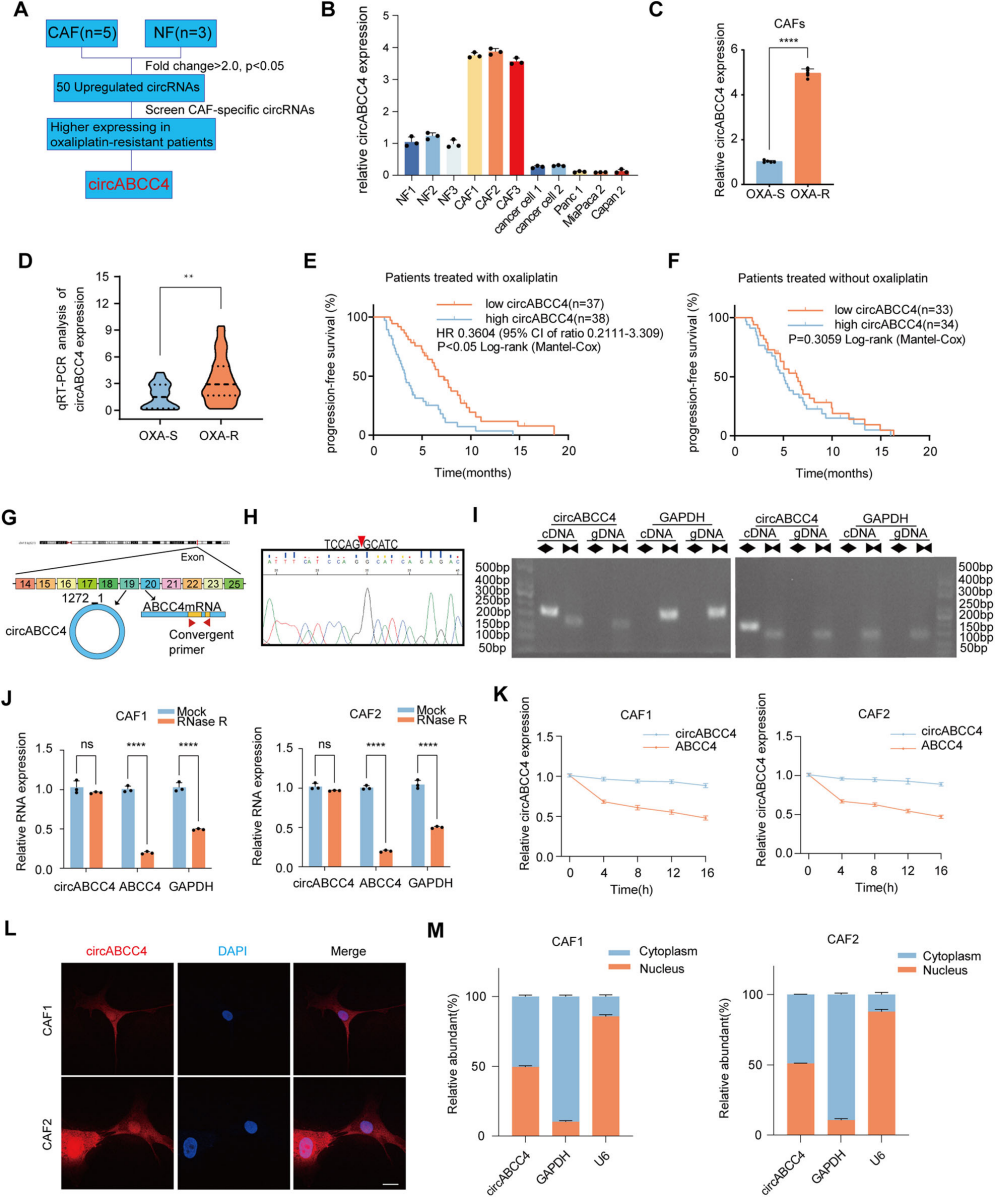
circABCC4 is overexpressed in CAFs and is associated with Oxaliplatin chemoresistance and poor survival in advanced PDAC. **A** Schematic illustration of the identification of circABCC4 upregulated in CAFs. **B** qRT–PCR analysis of circABCC4 expression in NFs, CAFs, primary cancer cell and PDAC cell lines. **C** Comparative analysis of circABCC4 expression in CAFs obtained from oxaliplatin-resistant (OXA-R) and -sensitive (OXA-S) pancreatic cancer patients via qRT–PCR (*n* = 3). **D** qRT-PCR evaluation of circABCC4 expression in OXA-S (*n* = 21) and OXA-R (*n* = 54) PDAC samples, depicted as violin plots. **E**, **F** Kaplan–Meier survival analysis for advanced PDAC patients receiving oxaliplatin-based (**E**) or non-oxaliplatin chemotherapy (**F**), stratified by high or low circABCC4 expression. **G** Genomic loci diagram depicting the exonic origin (11 exons) of circABCC4. **H** Validation of the back-splice junction of circABCC4 using Sanger sequencing. **I** Amplification of cDNA and gDNA in CAFs using convergent and divergent primers, with GAPDH as a negative control. **J** Comparative PCR analysis of circABCC4, ABCC4 and GAPDH expression in CAFs following RNase R treatment. Data are mean ± SD. ns, not significant. *p < 0.05, **p < 0.01, ***p < 0.001, ****p < 0.0001. **K** Temporal analysis of circABCC4 and ABCC4 mRNA expression in CAFs post-treatment with actinomycin (**D**). **L** Representative FISH images for circABCC4 in CAF1 and CAF2. Scale bars, 20 μm. **M** Subcellular fractionation assays of circABCC4 in CAF1 and CAF2. Data are expressed as the mean ± SD. ***p < 0.001.

To characterize circRNAs upregulated in CAFs mediating oxaliplatin resistance in PDAC, we first analyzed circRNA-seq data from CAFs and their paired normal fibroblasts (NFs) (GSE172096), identifying 50 upregulated circRNAs in CAFs (Fig. 1A). Subsequently, quantitative real-time polymerase chain reaction (qRT-PCR) revealed 10 circRNAs significantly upregulated in CAFs. Among these candidate circRNAs, we focused on circABCC4, formed by the circularization of ABCC4, a key gene associated with drug resistance. qRT-PCR revealed that circABCC4 was specifically highly expressed in CAFs (Fig. 1B). Further analysis revealed that circABCC4 levels were significantly elevated in CAFs from oxaliplatin-resistant PDAC patients compared to those sensitive to the treatment (Fig. 1C). Similarly, circABCC4 expression was elevated in bulk tumor samples from oxaliplatin-resistant patients compared to those from oxaliplatin-sensitive patients in a 75 late-stage PDAC cohort (Fig. 1D). Among patients receiving oxaliplatin treatment, individuals with higher circABCC4 expression exhibited poorer progression-free survival (PFS) compared to those with lower circABCC4 expression levels (p < 0.05). Notably, this association between circABCC4 expression and PFS did not extend to patients who did not receive oxaliplatin, underscoring the specificity of circABCC4’s role in mediating resistance to this particular chemotherapy (Fig. 1E, F).

circABCC4, comprising 11 exons from the ABCC4 gene and spanning 1272 nucleotides as per the circBase database (Fig. 1G), undergoes head-to-tail splicing, confirmed by Sanger sequencing (Fig. 1H). Nucleic acid electrophoresis revealed its amplification with specific primers in cDNA, not gDNA, indicating its circular nature in CAFs (Fig. 1I). After treated with RNase R and actinomycin D, circABCC4 showed potential resistance to RNase R and was more stable than linear ABCC4 mRNA (Fig. 1J, K). Fluorescence in situ hybridization (FISH) and cell fractionation assays showed that the distribution of circABCC4 was approximately equally in the cytoplasm and the nucleus (Fig. 1L, M).

### circABCC4 is critical for CAFs to induce oxaliplatin resistance in PDAC cells

**Fig. 2:**
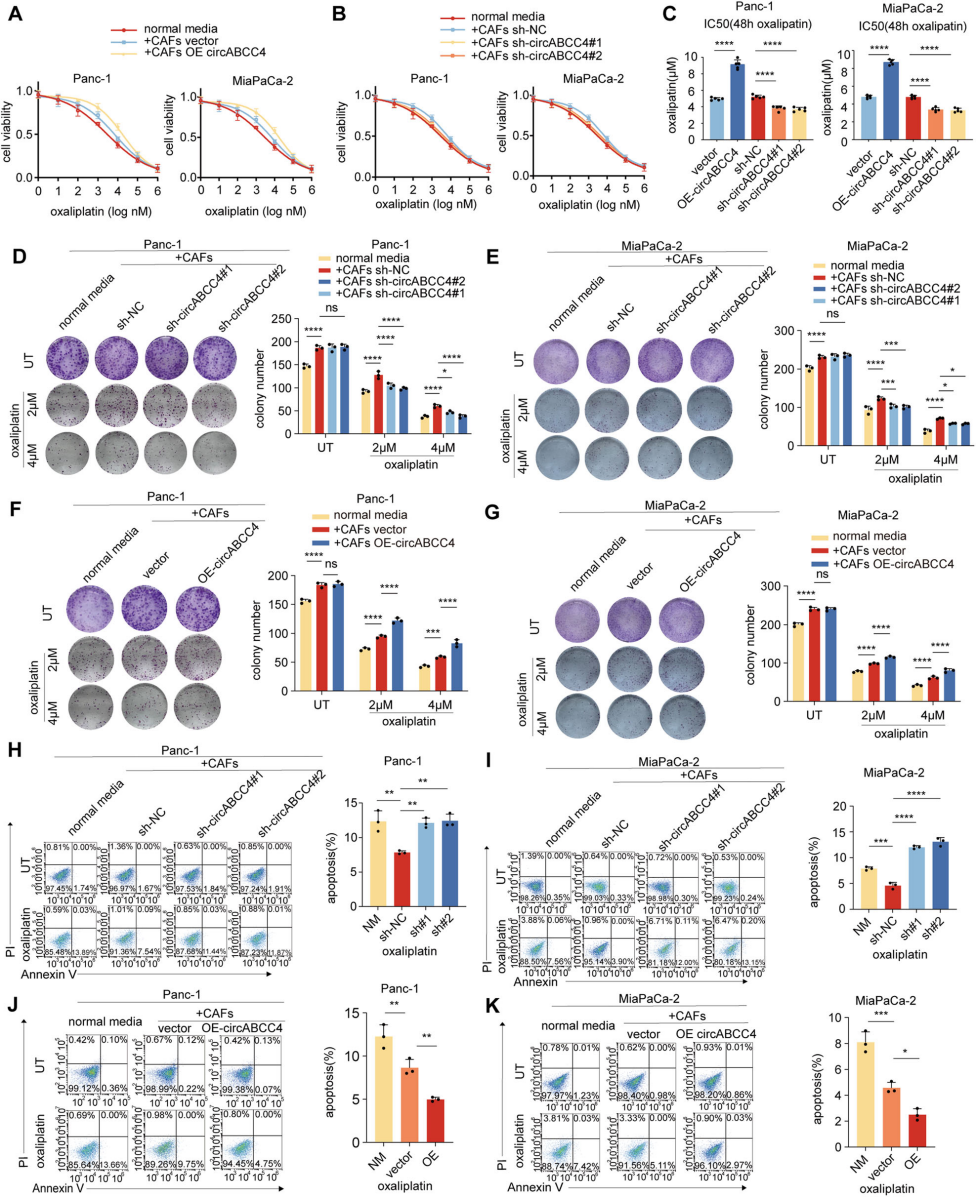
circABCC4 is critical for CAFs to induce oxaliplatin resistance in PDAC cells. Panc-1 and MiaPaCa-2 cells were cocultured with CAFs stably transfected with empty vector, circABCC4, lenti-NC-shRNA, or lenti-circABCC4-shRNA for 3 days and then subjected to the indicated experiments. **A**–**C** Oxaliplatin IC50 of Panc-1 and MiaPaCa-2 determined by constructing a dose–response curve. **D**–**G** Colony formation assays of Panc-1 and MiaPaCa-2 cells without or under oxaliplatin treatment (2 μM and 4 μM). **H**–**K** Flow cytometry analysis of oxaliplatin-induced (30 μM) apoptosis in Panc-1 and MiaPaCa-2 cells. Data are expressed as the mean ± SD. *p < 0.05, **p < 0.01, ***p < 0.001, ****p < 0.0001.

To investigate the role of circABCC4 in inducing oxaliplatin resistance in pancreatic cancer cells in vitro, we constructed a circABCC4 overexpression vector and two targeted shRNAs. Successfully introduced into CAFs, the overexpression vector did not affect the mRNA or protein levels of the parent ABCC4 gene, ensuring specific upregulation of circABCC4 (Fig. S1A-B). Similarly, shRNA-mediated silencing of circABCC4 did not impact the expression of ABCC4 (Fig. S1C). CCK-8 assays showed that CAFs transfected with circABCC4 overexpression vector increased the IC50 value of oxaliplatin by about two times in tumor cells, whereas circABCC4 knockdown markedly attenuated this effect (Fig. 2A–C). Silencing or overexpressing circABCC4 did not impact the number of clones of tumor cells without oxaliplatin. However, when treating tumor cells with oxaliplatin, plate cloning assays showed that the number of clones of tumor cells in the knockdown group decreased relatively (Fig. 2D, E). Conversely, the clone ability of tumor cell in circABCC4 overexpressing CAFs group increased significantly (Fig. 2F, G). Apoptosis assays aligned with these observations, revealing that circABCC4 overexpression in CAFs decreased apoptotic rates in tumor cells treated with oxaliplatin, an effect mitigated by circABCC4 silencing (Fig. 2H–K). Altogether, these results affirm that circABCC4 plays a pivotal role in mediating oxaliplatin resistance in pancreatic cancer cells through its expression in CAFs.

### circABCC4 affects the glycolysis of CAFs to influence oxaliplatin resistance in PDAC cells

**Fig. 3:**
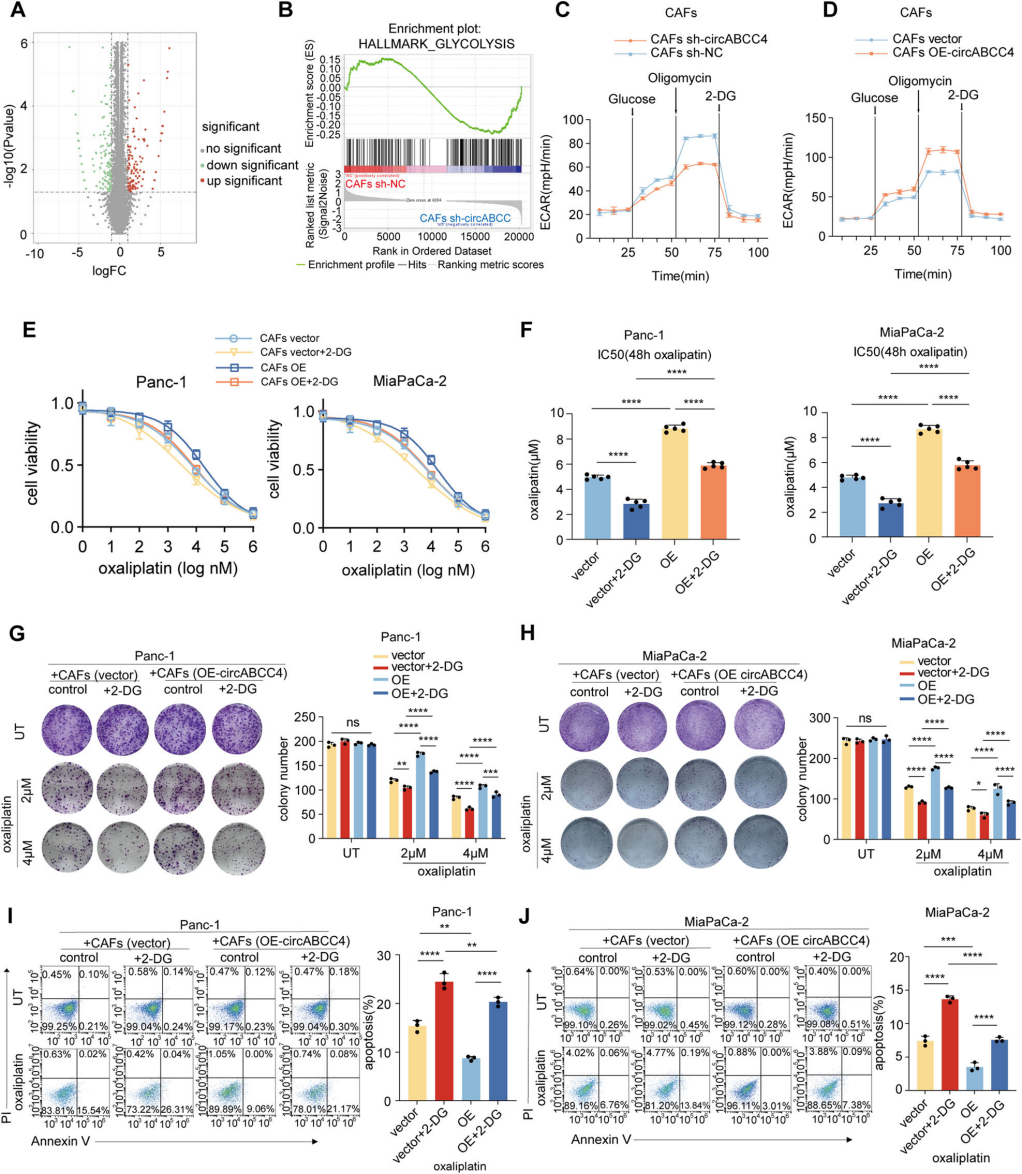
circABCC4 affects the glycolysis of CAFs, and inhibiting the glycolysis of CAFs will weaken oxaliplatin resistance in PDAC cells. Panc-1 and MiaPaCa-2 cells were cocultured with CAFs stably transfected with empty vector and circABCC4 for 3 days, and then subjected to the indicated experiments after adding 2-DG. **A** Plot showing the sums of the expression levels of genes regulated by circABCC4. **B** Glycolysis pathways was enriched of differential mRNA expression between CAFs and circABCC4-knockdown CAFs. **C**, **D** The extracellular acidification rate (ECAR) of circABCC4-silencing/overexpressing CAFs was monitored. **E**, **F** Oxaliplatin IC50 of Panc-1 and MiaPaCa-2 determined by constructing a dose–response curve. **G**, **H** Colony formation assays of Panc-1 and MiaPaCa-2 cells without or under oxaliplatin treatment (2 μM and 4 μM). **I**, **J** Flow cytometry analysis of oxaliplatin-induced (30 μM) apoptosis in Panc-1 and MiaPaCa-2 cells. Data are expressed as the mean ± SD. *p < 0.05, **p < 0.01, ***p < 0.001, ****p < 0.0001.

To elucidate how circABCC4 functions in CAFs, we conducted next-generation sequencing on CAFs transfected with sh-circABCC4 or control (Fig. 3A). Gene Set Enrichment Analysis (GSEA) indicated an enhancement of the glycolysis pathway in control CAFs, an effect diminished upon circABCC4 silencing (Fig. 3B). Silencing circABCC4 in CAFs showed a significant decrease in intracellular lactate levels, extracellular acidification rate (ECAR) (Fig. 3C, D) and glucose uptake rate (Fig. S2A, B), compared to the control group. To clary whether circABCC4 influenced glycolysis in CAFs to mediate oxaliplatin resistance, CAFs were pretreated with the glycolysis inhibitor 2-deoxyglucose (2-DG) and then co-cultured with pancreatic cancer cells. And the tumor cells were collected and treated with oxaliplatin. We found that treatment of 2-DG significantly abolished the effect of cicrABCC4 overexpression in enhancing IC50 value of oxaliplatin, cell cloning ability, and anti-apoptotic ability of pancreatic cancer cells. While, 2-DG did not impact the number of clones of tumor cells without oxaliplatin (Fig. 3E–J). Together, these findings underscore the critical involvement of circABCC4 within CAFs in driving glycolysis-dependent oxaliplatin resistance in pancreatic cancer.

### circABCC4 facilitates PKM2 nuclear translocation in CAFs to enhance oxaliplatin resistance in PDAC cells

**Fig. 4:**
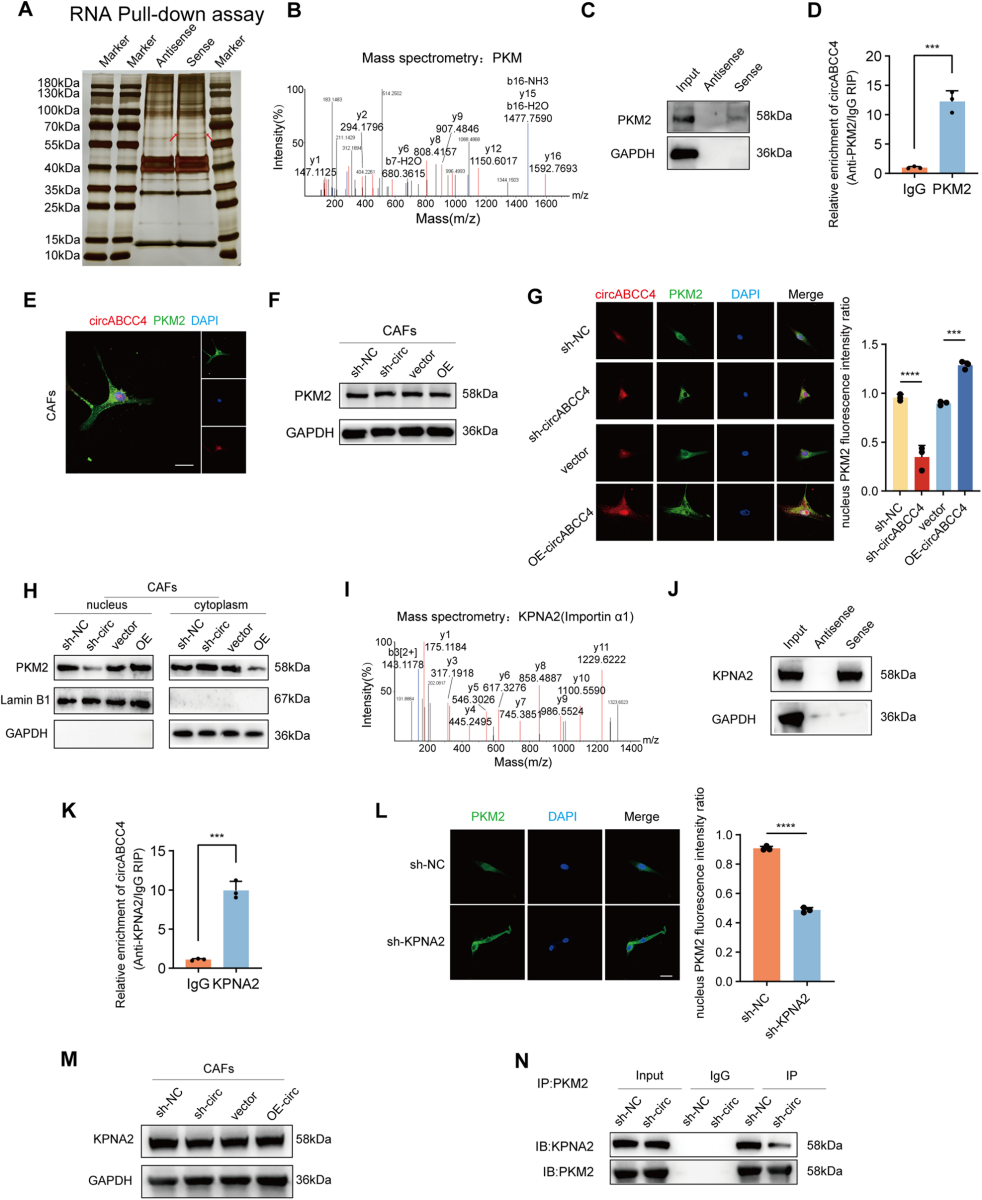
circABCC4 facilitates PKM2 nuclear translocation in CAFs. **A** Silver staining for RNA pull-down assay with the specific biotin-labeled circABCC4 probe in CAFs lysates. Red arrows indicate the unique differential band precipitated by the circABCC4 probe. **B**, **C** Mass spectrometry (**B**) and western blot (**C**) analysis of proteins in unique differential bands. PKM2 was identified as a candidate protein interacting with circABCC4. **D** RNA immunoprecipitation (RIP) assays in CAFs using IgG and PKM2 antibodies. The relative enrichment of circABCC4 was calculated by qRT-PCR. **E** Dual RNA-FISH and immunofluorescence staining assay indicating the colocalization of circABCC4 (red) and PKM2 (green), with nuclear staining with DAPI (blue). Scale bars, 40 μm. **F** Western blot of the expression kinetics of PKM2 in CAFs transfected with empty vector, circABCC4, lenti-NC-shRNA, or lenti-circABCC4-shRNA. **G** Dual RNA-FISH and immunofluorescence staining assay indicating the colocalization of circABCC4 (red) and PKM2 (green), with nuclear staining with DAPI (blue) in CAFs stably transfected with empty vector, circABCC4, lenti-NC-shRNA, or lenti-circABCC4-shRNA for 3 days. Scale bars, 40 μm. **H** Western blot of the expression kinetics of PKM2 in CAFs’ nucleus and cytoplasm, transfected with empty vector, circABCC4, lenti-NC-shRNA, or lenti-circABCC4-shRNA. **I**, **J** Mass spectrometry (**I**) and western blot (**J**) analysis of proteins in unique differential bands. KPNA2 (Importin α-1) was identified as a candidate protein interacting with circABCC4. **K** RNA immunoprecipitation (RIP) assays in CAFs using IgG and KPNA2 antibodies. The relative enrichment of circABCC4 was calculated by qRT-PCR. **L** Immunofluorescence staining assay indicating the localization of PKM2 (green) with nuclear staining with DAPI (blue) in CAFs. Scale bars, 40 μm. **M** Western blot of the expression kinetics of KPNA2 in CAFs transfected with empty vector, circABCC4, lenti-NC-shRNA, or lenti-circABCC4-shRNA. **N** Western blot of the expression kinetics of KPNA2 binding to PKM2 in CAFs in CAFs transfected with empty vector, circABCC4, lenti-NC-shRNA, or lenti-circABCC4-shRNA by immunoprecipitation assay.

To investigate the mechanism by which circABCC4 influences glycolysis in CAFs, we developed a biotinylated circABCC4 probe targeting its splicing sequence and conducted an RNA pulldown assay to identify interacting proteins within CAFs. Silver staining revealed a distinct band at 55–70 kDa enriched by the biotinylated circABCC4 probes (Fig. 4A). Mass spectrometry analysis along with western blotting indicated that PKM2 was enriched on circABCC4 (Fig. 4B, C). The interaction between circABCC4 and PKM2 was further confirmed by RNA immunoprecipitation (RIP) assay (Fig. 4D). While, PKM1 was not enriched on circABCC4(Fig. S3A, B). Alternative splicing of PKM results in the production of two variants: PKM1, which includes exon 9 of the PKM gene and is expressed in the majority of adult tissues, and PKM2, which contains exon 10 and is primarily found in fetal tissues and cancer cells. The amino acid sequence of exon10 after translation of PKM2 is in 389–433 aa [26]. We generated 4 HA-tagged truncations of PKM2 as outlined in a previous study [27]. RIP assay showed that 390-531 aa was the key region of PKM2 binding to circABCC4 (Fig. S3C). Thus, we regarded circABCC4 combined PKM2 in the specific region which is absent in PKM1. Fluorescence in situ hybridization (FISH) assays showed that circABCC4 and PKM2 co-localized in CAFs cells (Fig. 4E). Following circABCC4 overexpression or knockdown, total PKM2 protein levels in CAFs remained unchanged (Fig. 4F). Interestingly, we observed that the circABCC4 overexpression promoted the PKM2 nuclear translocation in CAFs, while circABCC4 depletion reversed this effect (Fig. 4G, H). We found karyopherin subunit alpha 2 (KPNA2), a member of the nuclear transport protein family, was enriched by circABCC4 in RNA pulldown assay. Mass spectrometry analysis along with western blotting indicated that KPNA2 was enriched on circABCC4 (Fig. 4I, J). The interaction between circABCC4 and KPNA2 was further confirmed by RIP (Fig. 4K). Knockdown of KPNA2 decreased PKM2 translocation to the nuclear (Fig. 4L). Upon overexpression or knockdown of circABCC4, no significant changes were observed in the levels of KPNA2 protein levels in the total protein of CAFs (Fig. 4H). Co-IP assay indicated that knockdown of circABCC4 resulted in a weakened interaction between PKM2 and KPNA2 (Fig. 4N, S3D). Together, our data indicated that circABCC4 enhanced the interaction of PKM2 and KPNA2 and facilitated the nuclear relocation of PKM2.

To assess the effect of PKM2 nuclear translocation on oxaliplatin resistance in pancreatic cancer, CAFs were pretreated with PKM2 nuclear translocation inhibitor (shikonin) and transfected with circABCC4, and then co-cultured with pancreatic cancer cells. Our results revealed that shikonin significantly counteracted the circABCC4 overexpression effects, reducing the IC50 value of oxaliplatin, diminishing cell cloning efficiency, and decreasing the anti-apoptotic capacity of pancreatic cancer cells. While, shikonin did not impact the number of clones of tumor cells without oxaliplatin (Fig. S4A–F).

### PKM2 nuclear translocation promotes glycolytic reprogramming of CAFs and affects DNA damage repair in PDAC cells

**Fig. 5:**
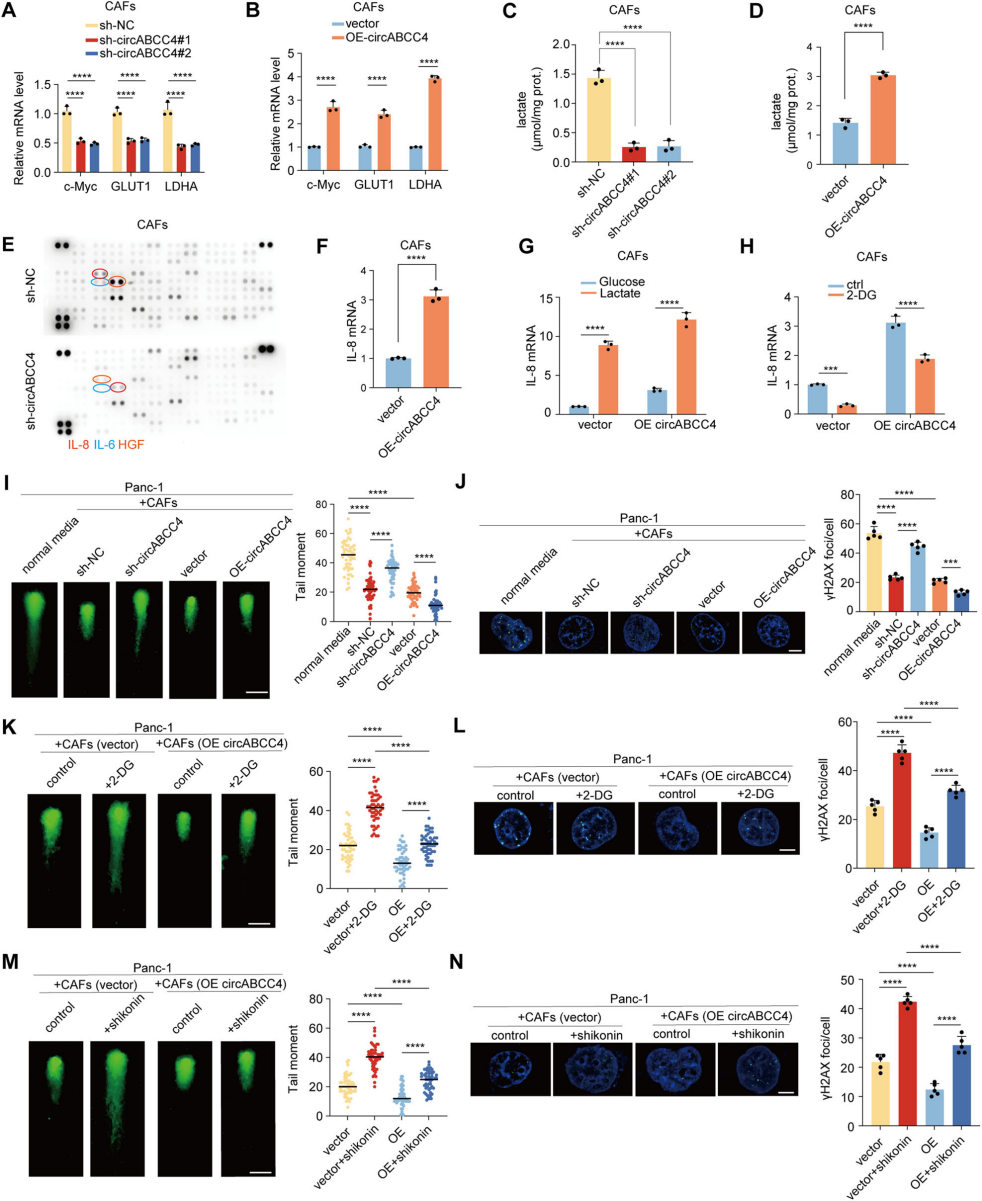
PKM2 nuclear translocation promotes glycolytic reprogramming of CAFs and affects DNA loss repair in PDAC cells. **A**, **B** The relative mRNA levels of c-Myc, GLUT1 and LDHA were measured by qPCR in CAFs cells transfected with empty vector, circABCC4, lenti-NC-shRNA, or lenti- NC-shRNA, or lenti-circABCC4-shRNA. **C**, **D** The lactate levels in CAFs transfected with empty vector, circABCC4, lenti-NC-shRNA, or lenti-circABCC4-shRNA were quantified. **E** Cytokine antibody array of conditional medium from CAFs transfected with lenti-NC-shRNA, or lenti-circABCC4-shRNA. **F**–**H** IL-8 mRNA expression determined by qPCR from CAFs transfected with empty vector or circABCC4, and then added with glucose, lactate (**G**) or 2-DG (**H**). **I** Imaging and quantification of oxaliplatin-induced DNA damage using a neutral comet assay from Panc-1 after coculturing with CAFs transfected with empty vector, circABCC4, lenti-NC-shRNA, or lenti-circABCC4-shRNA. Scale bar, 200 μm. **J** Imaging and quantification of γH2AX-positive foci from Panc-1 after coculturing with CAFs transfected with empty vector, circABCC4, lenti-NC-shRNA, or lenti-circABCC4-shRNA. Scale bar, 5 μm. **K** Imaging and quantification of oxaliplatin-induced DNA damage using a neutral comet assay from Panc-1 after coculturing with CAFs treated with 2-DG, Scale bar, 200 μm. **L** Imaging and quantification of γH2AX-positive foci from Panc-1 after coculturing with CAFs treated with 2-DG. Scale bar, 5 μm. **M** Imaging and quantification of oxaliplatin-induced DNA damage using a neutral comet assay from Panc-1 after coculturing with CAFs treated with shikonin, Scale bar, 200 μm. **N** Imaging and quantification of γH2AX-positive foci from Panc-1 after coculturing with CAFs treated with shikonin. Scale bar, 5 μm.

It is known that PKM2 nuclear translocation can promote cellular glycolysis [27]. To further evaluate the functional consequences of circABCC4-mediated PKM2 nuclear translocation, we measured the transcription levels of PKM2 target genes. qRT-PCR results indicated that silencing circABCC4 decreased the mRNA expression levels of c-Myc, GLUT1 and LDHA, while overexpressing circABCC4 increased the above mRNA expression levels (Fig. 5A, B). As c-Myc functions also as a major glycolytic regulator, we next explored the relationship among circABCC4, PKM2 translocation and c-Myc. qRT-PCR results indicated that the inhibition of PKM2 translocation could also reduce the expression of c-Myc, GLUT1 and LDHA (Fig. S5A). Luciferase reporter assays showed increased luciferase activity in the c-Myc promoter in CAFs overexpressing PKM2 compared with the vector group (Fig. S5B). Then, plate cloning assays showed the number of clones of tumor cells in the circABCC4 knockdown group decreased more significantly than the c-Myc knockdown group (Fig. S5C, D). Apoptosis assays revealed that circABCC4 knockdown in CAFs showed larger apoptotic rates than c-Myc knockdown (Fig. S5E, F). Subsequently, we determined whether circABCC4 affects the glycolytic flux of CAFs. Silencing circABCC4 showed a significant decrease in intracellular lactate levels in CAFs, while overexpressing circABCC4 enhanced lactate production in CAFs (Fig. 5C, D). It is reported that glycolysis activation of CAFs stimulate cells to secrete sorts of cytokines [28]. Protein microarray analysis revealed that circRNA alterations modulate the cytokine secretion profile of CAFs, with the secretion of IL-8 showing the most significant changes (Fig. 5E). qRT-PCR and ELISA further confirmed that overexpression of circABCC4 upregulated the expression and secretion of IL-8. Treatment with lactate significantly enhanced the circABCC4-mediated upregulation of IL-8; while, inhibition of glycolysis significantly reversed the circABCC4-mediated upregulation of IL-8. (Fig. 5F–H). Meanwhile, treatment with more lactate enhanced more circABCC4-mediated upregulation of IL-8 (Fig. S5G). Our previous research has elucidated that IL-8 derived from CAFs can promote platinum resistance in pancreatic cancer through DNA damage repair [15]. Comet assay showed that the comet tails lengths of tumor cells co-cultured with CAFs were shorter than those of tumor cells single cultured in regular medium. Knocking down circABCC4 weakened the effect of CAFs in shortening the comet tail length of tumor cells, while overexpressing circABCC4 promoted this effect. (Fig. 5I). Similarly, knocking down circABCC4 attenuated the capability of CAFs in reducing the γH2AX levels in tumor cells, while overexpressing circABCC4 promoted this effect (Fig. 5J). Treating CAFs with 2-DG or shikonin reversed the effects driven by circABCC4 in both shortening the comet tail length of tumor cells and lowering γH2AX levels in tumor cells (Fig. 5K–N). In summary, circABCC4 mediated the activation of glycolysis in CAFs by promoting PKM2 nuclear translocation, resulting in reducing DNA damage in tumor cells and consequently leading to oxaliplatin resistance.

Recently, it has been discovered that lactate, a glycolytic intermediate, can promote the activation of the NF-kB pathway to increase secretion of IL-8 [29]. Western blot suggested that overexpression of circABCC4 could increase the expression of p-IκBα, p-P65, and IL-8, while the expression of IκBα decreased correspondingly, while knockdown of circABCC4 showed the opposite result (Figure S6A). Moreover, inhibition of glycolysis or PKM2 nuclear translocation significantly reduced the expression of p-IκBα, p-P65, and IL-8 in CAFs (Figure S6B-C).

### circABCC4 regulates oxaliplatin resistance induced by CAFs in mice

**Fig. 6:**
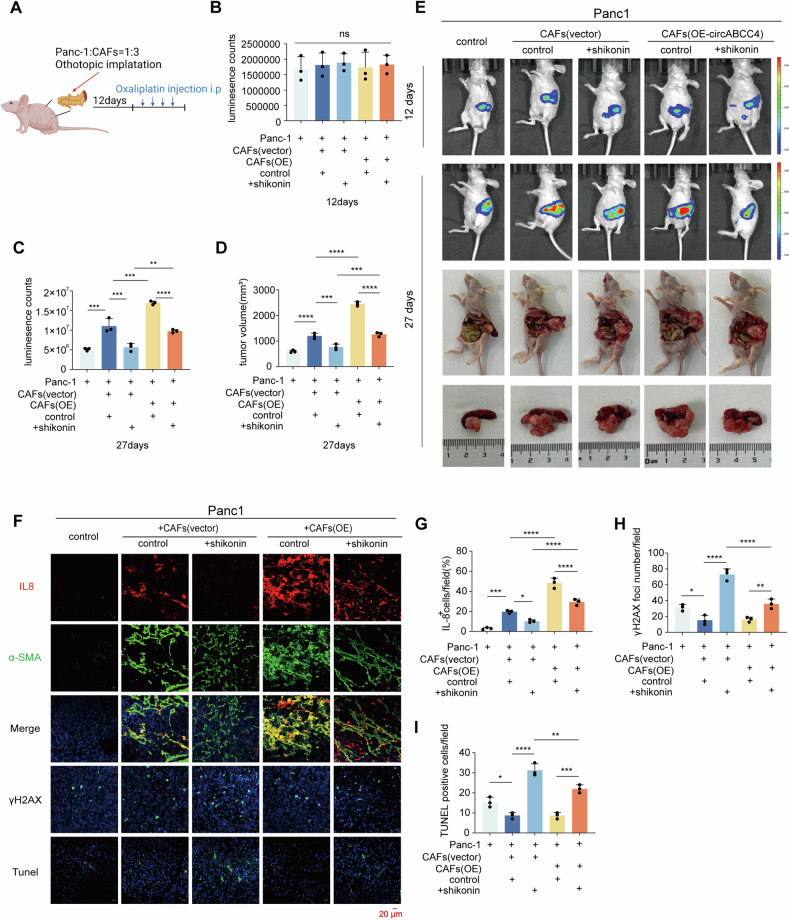
circABCC4 regulates oxaliplatin resistance induced by CAFs in mice. **A** Once 12 days after implantation, xenograft orthotopic model receiving oxaliplatin (5 mg/kg) once every 3 days. **B**, **C** Analysis of luminescence intensity in orthotopic xenograft model. The relative luminescence intensity = (Xday27-Xday12)/average (shNegday27-shNegday12). **D** Analysis of orthotopic tumor volume. The results are presented as the mean ± SD. *N* = 5/per group, *P < 0.05, **P < 0.01, ***P < 0.001, ****P < 0.0001. ns no significance. **E** Representative IVIS images and pancreatic tumors in orthotopic xenograft model. **F** Representative images of immunofluorescence analysis of IL-8, α-SMA, γH2AX and TUNEL assays of orthotopic model. **G**, **H** Quantification of the presence of IL-8^+^ (**G**), the number of γh2ax-positive foci (**H**) and TUNEL-positive cells (**I**) in each group.

To explore the role of circABCC4 in vivo, we established a xenograft orthotopic model. Panc-1 cells were co-injected with or without CAFs into the pancreas of nude mice (Fig. 6A). All mice were treated with oxaliplatin after 12 days. On the 12th day, all groups initially showed similar levels of IVIS (in vivo imaging system) signals (Fig. 6B, E). On the 27th day, the group of mice co-injected with CAFs showed a stronger IVIS signal, larger tumor sizes, compared to the group injected without CAFs. These effects were enhanced by overexpressing circABCC4 in CAFs and reversed by treatment with shikonin (Fig. 6C–E). Additionally, immunofluorescence staining assays demonstrated that co-injection with CAFs caused higher IL-8 level, lower γH2AX and Tunel level compared to injection with Panc-1 alone. Overexpressing circABCC4 in CAFs increased IL-8 level and decreased γH2AX and Tunel level, while treatment with shikonin reversed these effects (Fig. 6F–I). In total, circABCC4 in CAFs induced oxaliplatin resistance via PKM2 nuclear translocation in vivo.

### circABCC4 is associated with DNA repair and oxaliplatin resistance in PDAC patients

**Fig. 7:**
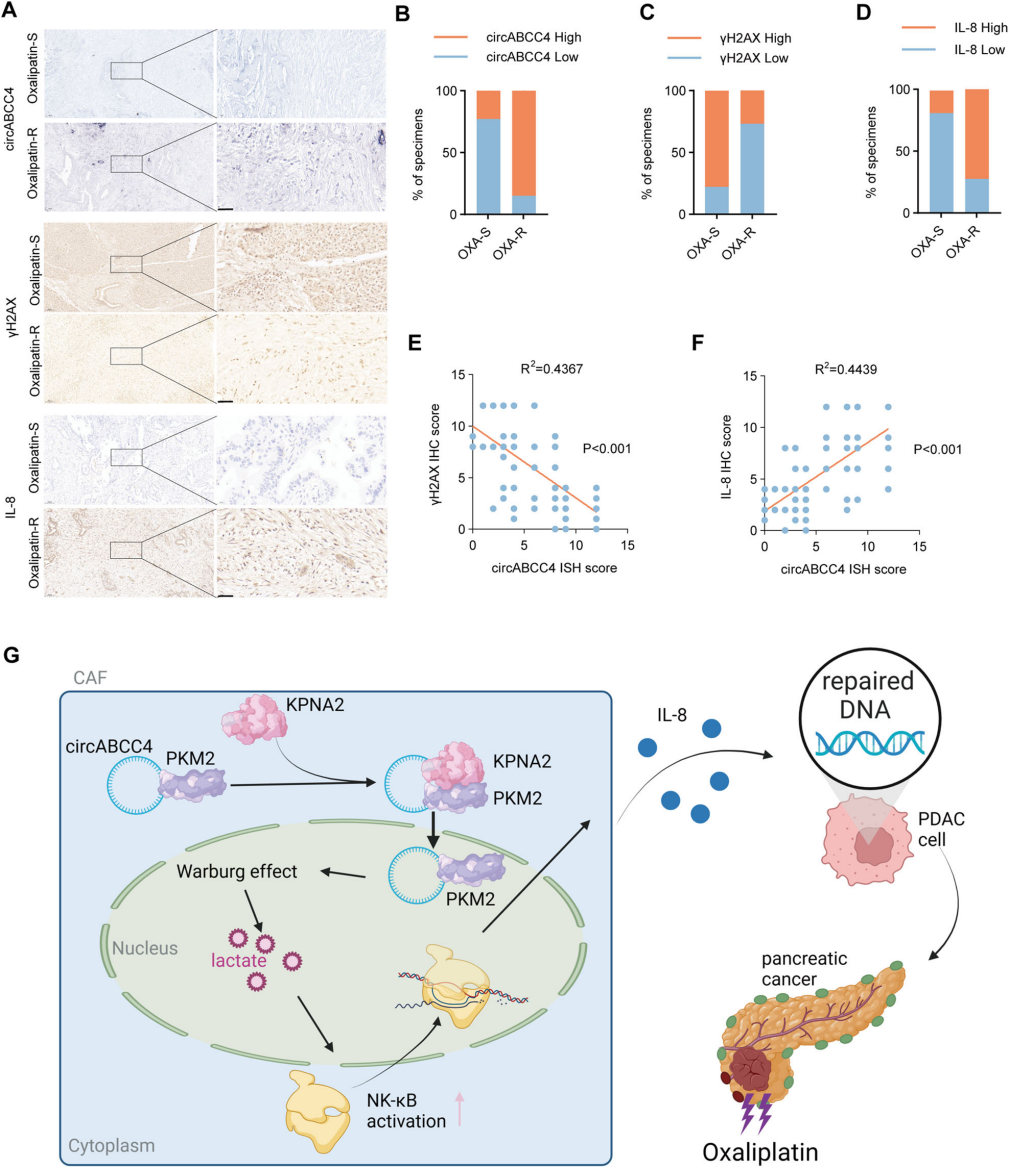
circABCC4 is associated with DNA repair and oxaliplatin resistance in PDAC patients. **A** Depiction of representative ISH results for circABCC4 and IHC for IL-8 and γH2AX in oxaliplatin-resistant (OXA-R, *n* = 54) and oxaliplatin-sensitive (OXA-S, *n* = 21) pancreatic cancer samples. Scale bars, 40 μm. **B**–**D** Quantitative breakdown of the specimen percentages featuring low or high expression of circABCC4 (B), γH2AX (C) and IL-8 (**D**) across oxaliplatin-resistant and oxaliplatin-sensitive groups. **E** Correlation between circABCC4 expression and γH2AX protein levels in PDAC tissues. **F** Correlation between circABCC4 expression and IL-8 protein levels in PDAC tissues. **G** Graphical illustration of circABCC4 from CAFs mediating PKM2 to promote oxaliplatin resistance in PDAC cell.

To evaluate the predictive value and clinical significance of circABCC4 in predicting the response to oxaliplatin chemotherapy, we examined the expression of circABCC4 in primary tumor tissues of 75 advanced PDAC patients receiving oxaliplatin chemotherapy, and assessed the level of DNA damage of γH2AX through immunohistochemical staining. In oxaliplatin-resistant patients, compared to those who responded well to oxaliplatin treatment, we observed an increase in circABCC4 levels and a decrease in γH2AX levels in tumor tissues (Fig. 7A–C). circABCC4 expression is negatively correlated with γH2AX expression (Fig. 7D). Furthermore, in oxaliplatin-resistant patients, we observed an increase in IL-8 levels in tumor tissues, as well as we observed a positive correlation between the levels of IL8 and circABCC4 expression. (Figure 8E-F). In conclusion, our research results suggest that circABCC4 may serve as a potential biological marker for oxaliplatin chemotherapy resistance in pancreatic cancer (Fig. 7H).

## Discussion

Chemotherapeutic resistance in pancreatic cancer presents a significant challenge, limiting the efficacy of current treatment modalities and impacting patient prognosis [30, 31]. Notably, the advent of targeted therapies, such as olaparib, for BRCA-mutated genes has underscored the importance of understanding mechanisms underlying platinum drug sensitivity and resistance [11, 32]. Among these mechanisms, TME has emerged as a pivotal factor in modulating chemoresistance, particularly to platinum-based therapies [13]. Due to the pronounced desmoplastic response observed in pancreatic cancer, the abundant presence of CAFs within the tumor stroma plays a pivotal role in mediating chemoresistance in this densely fibrotic malignancy [33]. Our recent study revealed that CAFs trigger oxaliplatin resistance in pancreatic cancer via IL-8 activation, leading to increased lncRNA UPK1A-AS1 and enhanced NHEJ DNA repair, underscoring ncRNAs vital role in TME-driven platinum resistance [15]. However, the specific mechanisms through which lncRNAs influence CAFs behavior to foster resistance are yet to be elucidated. In our research, we identified a crucial elevation of circABCC4 within CAFs, instrumental in promoting oxaliplatin resistance in PDAC. This form of resistance arises from the glycolytic reprogramming within CAFs, which boosts IL-8 secretion towards PDAC cells, enhancing their resistance to platinum-based therapies. Central to this process, circABCC4 in CAFs facilitates the nuclear translocation of PKM2, enhancing glycolytic reprogramming and bolstering NHEJ DNA repair in PDAC cells. This study breaks new ground by revealing how circRNA modulates glycolysis in CAFs to drive oxaliplatin resistance in pancreatic cancer. By spotlighting the circABCC4/PKM2/IL8 pathway, we introduce a promising strategy to overcome platinum resistance, significantly advancing potential treatments.

Emerging evidence suggests that the altered physical properties of the TME can lead to widespread epigenomic changes, fostering the selection of traits that enable cancer cells to clonally expand and enhanced drug resistance [34]. Recent discoveries have started to shed light on how circRNAs contribute to epigenetic alterations in pancreatic cancer [35], though their specific mechanisms in reshaping the tumor microenvironment remain largely unexplored. Our novel finding that extracellular vesicle-packaged circBIRC6, originating from CAFs, enhances platinum resistance through SUMOylation, modulation, highlighting the pivotal role of CAFs-derived circRNAs in chemotherapy resistance [13]. Building on our initial RNA-seq analysis of CAFs and NFs (GSE172096), with a focus on circRNAs highly expressed in CAFs but not secreted via extracellular vesicles, we identified a novel circRNA, circABCC4, that influences CAFs metabolism, thereby enhancing platinum resistance in pancreatic cancer cells. A recent study found that the circRARS/IGF2BP3 complex enhances m6A recognition on specific genes, thereby regulating lipid metabolism to foster sunitinib resistance in renal cell carcinoma [36]. Qi et al. recently revealed that CAFs confer chemoresistance by modulating ferroptosis-associated metabolic pathways via ncRNA, particularly through the miR-3173-5p/ACSL4 pathway, highlighting a novel therapeutic approach by targeting TME-derived ncRNAs to curb metabolic-driven drug resistance [37]. Our research pioneers in linking circABCC4 levels in CAFs with oxaliplatin sensitivity in PDAC patients, unveiling the potential of circABCC4 as a therapeutic target within the TME to bypass platinum resistance in PDAC.

Our research explores the role of circABCC4 in enhancing oxaliplatin resistance in pancreatic cancer by elucidating its influence on glycolysis within CAFs. A recent study in soft-tissue sarcomas identified a novel subset of CAFs, known as glycolytic CAFs (glyCAFs), which hinder T-cell infiltration through GLUT1-CXCL16 signaling, indicating that targeting glycolysis in CAFs could boost T-cell infiltration and enhance chemotherapy outcomes [17]. A previous study shown that LINC00092, activated by CXCL14-rich CAFs, drives ovarian cancer metastasis by modulating glycolysis via PFKFB2 interaction, underscoring a vital metabolic feedback loop between CAFs and cancer cell advancement [38]. However, the mechanisms by which circRNAs regulate glycolysis in CAFs to induce platinum resistance in tumors remain largely unexplored. Mans et al. found that CAFs displayed an elevated oxidative state and significantly heightened resistance to oxaliplatin, highlighting that targeting metabolism can effectively counteract pancreatic cancer progression [39]. Our study is the first to report that circABCC4 activates glycolysis in CAFs, affecting oxaliplatin resistance in PDAC cells, unveiling a novel strategy for targeting ncRNA-associated metabolism to overcome platinum-based drug resistance in tumors.

To elucidate how circABCC4 contributes to platinum resistance, we analyzed its associated proteome, identifying PKM2 as the key interacting protein. Recent research in triple-negative breast cancer (TNBC) has shown that PRMT1-induced methylation of PKM2, together with PHGDH and PFKFB3, shifts glucose metabolism to serine synthesis, increasing fatty acid production and chemoresistance, underscoring the crucial role of PKM2 modifications in tumor drug resistance [40]. Interestingly, our study unveils for the first time that circABCC4 directly interacts with PKM2, which then attracts the essential transporter KPNA2, leading to the enhanced nuclear import of PKM2. Wu et al. reveals that inhibiting PKM2 nuclear translocation in CAFs through TGF-βRII overexpression crucially disrupts glycolytic activation, uncovering the critical function of PKM2 localization in CAFs-mediated metabolic reprogramming and suggesting a therapeutic target for oral cancer [41]. Consistently, our research demonstrates that circABCC4-mediated nuclear translocation of PKM2 activates aerobic glycolysis in CAFs, which in turn stimulates the NF-kB pathway via the lactate route, thereby promoting platinum resistance in pancreatic cancer cells through an IL-8 dependent mechanism. Previous research has shown that inducing PKM2 tetramerization significantly inhibits its nuclear translocation, thereby reducing glycolysis-driven chemoresistance [42]. Notably, in vivo experiments demonstrated that inhibiting the nuclear translocation of PKM2 markedly decreases oxaliplatin resistance, suggesting targeting circABCC4 to suppress PKM2-related chemoresistance offers a novel therapeutic strategy. Our prior findings reveal that activated inflammatory CAFs (iCAFs) enhance oxaliplatin resistance in pancreatic cancer through an IL-8-driven DNA damage repair pathway [15], yet the activation mechanism of IL-8 by iCAFs is still to be clarified. Our research unveils that circABCC4 triggers glycolysis, leading to IL-8 secretion via the NF-kB pathway, highlighting a potential pivotal role of circABCC4 in activating iCAFs. We aim to delve deeper into how circABCC4 drives the transformation among CAFs subtypes and the heterogeneity of glycolytic activation across these subgroups in future investigations.

In translating our findings to a clinical setting, we explored the prognostic significance of circABCC4 in PDAC patients. Our research identified a negative association between circABCC4 levels and γH2AX, suggesting tumors with elevated circABCC4 exhibit a weaker DNA damage response. Further, patients showing higher circABCC4 levels from CAFs experienced poorer responses to oxaliplatin chemotherapy, highlighting the impact of circABCC4 on treatment efficacy. Our earlier studies indicate a significant correlation between serum IL-8 levels and both the prognosis and sensitivity to platinum-based chemotherapy in PDAC patients [15]. In our study, we observed a concurrent increase in circABCC4 and IL-8 expression in patients resistant to oxaliplatin, suggesting that combining tissue circABCC4 and serum IL-8 measurements may enhance the predictive accuracy of responses to platinum-based chemotherapy. Our study not only highlights the role of circABCC4 in forecasting PDAC outcomes but also positions it as a key indicator for assessing responsiveness to platinum-based chemotherapy in PDAC patients.

In summary, our study reveals a novel pathway by which the dynamic interaction between CAFs and PDAC cells fosters oxaliplatin resistance, casting a spotlight on the distinctive role of the noncoding RNA, circABCC4, in this process. By delineating the manner in which circABCC4 modulates PKM2 and activates glycolysis within CAFs, our research identifies this molecular interaction as a strategic target to bolster the effectiveness of platinum-based chemotherapy in PDAC, thereby advancing the frontier of cancer therapeutics.

## Methods

### Patient information and sample acquisition

Tissue specimens were gathered from a cohort of 75 advanced pancreatic ductal adenocarcinoma PDAC patients who received their primary platinum drug-based chemotherapy treatment at Sun Yat-Sen Memorial Hospital and Guangdong Provincial People’s Hospital between 2016 and 2021. Prior to this initial platinum drug-based chemotherapy regimen, these patients had not undergone any other forms of chemotherapy, radiation therapy, immunotherapy, or targeted therapy. Patients specifically underwent platinum-based chemotherapy following one of the three regimens: mFOLFIRINOX, characterized by an administration of oxaliplatin, irinotecan, leucovorin on the first day and a continuous 46 hour infusion of 5-FU across the first two days, recurring every two weeks; GemOx, entailing an administration of gemcitabine on days 1 and 8, and oxaliplatin on day 1, in cycles of three weeks; or GP, which consisted of gemcitabine on days 1 and 8, and cisplatin on day 1, repeating every three weeks. The therapeutic outcomes were assessed in accordance with the Response Evaluation Criteria in Solid Tumors (RECIST), version 1.1. At the four-month mark post the commencement of chemotherapy, patients showing Complete Response (CR) or Partial Response (PR) were categorized as chemosensitive, while those with Stable Disease (SD) or Progressive Disease (PD) were labeled as chemoresistant. Progression-free survival (PFS) was ascertained as the duration from the initiation of chemotherapy to the onset of disease progression.

### Primary human CAFs isolation and culture

CAFs lines were derived from PDAC tumor tissue. Freshly harvested tissues were mechanically and enzymatically dissociated using a tumor dissociation kit (Cat #130-095-929, Miltenyi Biotec, Bergisch Gladbach, Germany) on a gentleMACS™ Dissociator (Miltenyi Biotec). Briefly, the tissues were minced and digested into single-cell suspensions. After filtration with 70 mm cell strainers, the stromal fraction was collected by centrifugation at 250 g for 5 min and incubated with DMEM supplemented with 15% FBS. Magneticactivated cell sorting with anti-FSP (fibroblast-specific protein) was used to purify the primary human CAFs isolated as indicated above. CAFs were maintained in fibroblast medium (FM, ScienCell).

### Cells co‑culture

Approximately 1.5 × 10^5^ stably transfected CAFs were propagated in a co-culture dish for adherent growth. Then, approximately 5 × 10^4^ PDAC cell (Panc-1, MiaPaCa-2) were cultured in each six-well plates under the co-cultured dish with treated CAFs for 3 days.

### Cell lines

PDAC cell lines (Panc-1 and MiaPaCa-2) were obtained from the American Type Culture Collection (ATCC) and were maintained in Dulbecco’s modified eagle medium (DMEM, Gibco) supplemented with 10% fetal bovine serum (FBS, Gibco).

### RNA extraction and quantitative real‑time PCR (qRT‑PCR)

Total RNA from frozen tissues and cultured cell lines was extracted using Universal RNA Purification Kit (EZBioscience) and was measured using a NanoDrop 2000 spectrophotometer (Thermo Fisher Scientific, USA). After isolation, RNA was converted into complementary DNA (cDNA) using HiS cript Reverse Transcriptase (R101-01, Vazyme, China). Real-time quantitative PCR (qRT-PCR) was carried out with TB Green Premix Ex TaqTM kit (Takara, Japan) using the Light Cycler 480 detection system (Roche, Switzerland) with the following conditions: 95 °C, 10 min; (95 °C, 10 s; 60 °C, 10 s; 72 °C, 15 s) x 45 cycles. The 2^-∆∆Ct^ method was used to analyze the relative expression levels of target gene. The specific primer sequences employed in the qRT-PCR analyses are enumerated in Table S1.

### RNase R digestion and actinomycin D assay

In the RNase R digestion assay procedure, total RNA that was extracted from NFs and CAFs underwent treatment with or without 5 U/µg of RNase R (RNR07250, Epicenter Technologies) and was subsequently incubated for a period of 30 min at 37 °C. In the actinomycin D assay, cells were exposed to 2 µg/mL of actinomycin D (Sigma, USA) at specific intervals that spanned from 0 to 24 h. Following this, the technique of qRT‑PCR was utilized to ascertain the expression levels of circABCC4 and ABCC4. Every experiment was independently conducted on three separate occasions to affirm its reliability and reproducibility.

### Cell transfection

The pCD-ciR vector was used by IGE (Guangzhou, China) to clone the entire sequence of circABCC4. The shRNAs targeting the loop-forming site of circABCC4 were obtained from IGE. The shRNA constructs, specifically designed for human circABCC4, were purchased from IGE. The shRNA sequences are included in Table S2.

### Western blot analysis

Add protease inhibitor (Abcam, ab141032) to RIPA buffer (Thermo Fisher Scientific, 89900) and use it to extract total proteins from cells. According to the manufacturer’s instructions, use the Total Protein Extraction Kit for animal cell and tissue samples (Invent Biotechnologies, SD-001/SN-002) to extract total proteins from tissue samples.

Load equivalent amounts of proteins onto SDS-polyacrylamide gels, perform electrophoresis, and transfer them to a polyvinylidene fluoride (PVDF) membrane. Block the membrane in 5% BSA for 1 h, then incubate overnight at 4 °C with primary antibodies. Subsequently, incubate the membrane with HRP-conjugated secondary antibodies at room temperature for 1 h. Visualize antigen-antibody reactions using enhanced chemiluminescence (ECL, Thermo Fisher Scientific) and crop the images according to the molecular weight markers of proteins (kDa) presented. Used antibodies are cataloged in Table S3.

### Cell counting kit-8 assay

For the evaluation of PDAC cell drug response, treated pancreatic cells were seeded at a density of 4000 cells per well in 96-well plates. The cells were then treated with oxaliplatin at concentrations ranging from 0 – 1000 µM for 72 hours. After incubation with 10 µl of CCK-8 solution (K1018, APExBIO, USA) at 37 °C for 2 hours, the absorbance at 450 nm was measured using a Tecan microplate reader. The drug response was determined based on the half-maximal inhibitory concentration (IC50) values calculated via GraphPad Prism 8.0. Cell viability for cell proliferation was assessed daily by measuring the absorbance at 450 nm.

### Colony formation assay

700 Panc-1 or MiaPaCa-2 cells were subjected to specific treatments and seeded into 6-well plates. They were allowed to attach for 24 h and then treated with fresh complete medium supplemented with 2 µM and 4 µM oxaliplatin for PDAC cells. After a 24 h exposure, the cells were cultured with fresh complete medium for 2 weeks. Colonies were fixed, stained, and manually counted, with three independent repetitions.

### Annexin V-PI apoptosis assay

Annexin V/PI staining (BMS500FI, Invitrogen, USA) was employed for the flow cytometry-based detection of cell apoptosis. Briefly, PDAC cells were seeded and transfected in 6-well plates. After co-culturing with transfected CAFs for 72 h, Panc-1 and MIAPaCa-2 cells were treated with 30 μM oxaliplatin for 48 h. Then the PDAC cells were collected, washed with PBS, and resuspended in a binding buffer. Subsequently, the cells were incubated with Annexin V-FITC and PI at room temperature in the dark for 15 min, followed by flow cytometric analysis within 1 h. The experiment was repeated three times. Flow cytometric analysis was performed using a flow cytometer (CytoFLEX S, Beckman Coulter), and the data were analyzed using FlowJo V10 (v.10.0.7r2).

### RNA pull‑down assay

RNA pull-down assays were performed by using a Magnetic RNA-Protein Pull-Down Kit (20164, Thermo Scientific, USA). The sense and anti-sense probes, targeting the binding sites of circABCC4, were synthesized by IGE (Guangzhou, China) and incubated with magnetic beads overnight at 4 °C. The RNA-binding protein complexes were then washed, eluted from the magnetic beads, and detected by Western blotting. Silver staining was performed using the Pierce Silver Stain kit (24612, Thermo Scientific, USA), and differential protein bands were excised and identified by mass spectrometry. Anti-sense probes are cataloged in Table S2.

### RNA sequencing

Total RNA was extracted and purified using TRIzol (Life Technologies, USA) following the standard protocol provided by the manufacturer. Following quality assessment with the Agilent 2100 Bioanalyzer (Agilent Technologies, USA) and NanoPhotometer (Implen, Germany), ribosomal RNA was depleted from 1 µg of total RNA. The construction of circRNA libraries was performed using the VAHTS Universal V6 RNA-seq Library Prep Kit for Illumina (Vazyme, China), following the manufacturer’s guidelines. Subsequently, each library was sequenced on an Illumina NovaSeq 6000 system (Illumina Corporation, USA) using the 150PE mode, according to the recommended protocol by Guangzhou Huayin Health Medical Group CO.,Ltd. (Guangzhou, China).

### Neutral comet assay

To assess DNA damage, Panc-1 and MiaPaCa-2 cells were treated with 50 µM and 30 µM oxaliplatin, respectively, for one hour before being harvested at specific time points. The neutral comet assays were performed using a Comet Assay Kit (Trevigen, USA), following the manufacturer’s instructions. The samples were then stained with SYBR Gold (Invitrogen, USA) to visualize the DNA. Observation and imaging of the DNA were conducted using an Olympus FluoView 500 microscope. To quantitatively evaluate the extent of DNA damage, the tail DNA content was analyzed using the CASP software.

### Immunofluorescence

Immunofluorescence was performed as previously described. Briefly, cells grown on confocal dishes were treated with oxaliplatin and then harvested. Post fixation and permeabilization, confocal dishes were treated with antibodies against γH2AX (ab81299, abcam, UK) and PKM2 (15822-1-AP, proteintech, USA) overnight at 4 °C. The confocal dishes were then rinsed, treated with Alexa Fluor 488-conjugated secondary antibodies, counterstained with DAPI, and visualized via confocal fluorescence microscopy (Carl Zeiss AG, Germany).

### RIP assays

Following the manufacturer’s instructions, cell lysates were incubated with target antibodies or negative control normal mouse IgG. The EZ-Magna RIP kit (17-700, Millipore, USA) is then used to separate and purify the enriched RNA from the magnetic beads. RIP assays are performed using qRT-PCR. The antibodies used in this study are listed in Table S3.

### Co-Immunoprecipitation (Co-IP)

Following the manufacturer’s instructions, CO-IP detection was performed using the Pierce Co-IP Kit (26149, Thermo Scientific, USA). Treated CAFs were collected and lysed in IP buffer. Ten micrograms of PKM2, KPNA2 or control IgG were added to the resin and mixed with 500 μg of protein mixture. The mixture was gently rotated overnight at 4 °C. After three washes, total proteins were extracted for Western blot analysis. The antibodies used in this study are listed in Table S3.

### Nucleoplasma separation

The PARIS kit (AM1921, Thermo Scientific, USA) was used to separate the CAFs’ cytoplasmic and nuclear RNAs and protein according to the manufacturer’s protocol. RNAs were then examined by qRT-PCR to calculate the ratio of circABCC4 in cytoplasmic and nucleus. The protein expression in cytoplasmic and nuclear were examined by Western blot analysis.

### Fluorescence in situ hybridization (FISH)

FISH was conducted with an In Situ Hybridization Kit (Gene Pharma, Guangzhou, China) following the manufacturer’s instructions. Cy3-labeled circABCC4(Gene Pharma, Guangzhou, China) were hybridized with cells overnight at 37 °C. All images were captured by confocal microscopy. The targeted sequences of probes are provided in Supplementary Table S2.

### Cytokine antibody array

A cytokine antibody array was performed using the Proteome Profiler Human XL Cytokine Array Kit (ARY022B, R&D Systems, USA). In this assay, the CAFs medium was incubated with an array membrane overnight at 4 °C. Then, the membrane was incubated with detection antibody cocktails for 2 h, followed by incubation with streptavidin-HRP for 1 h. Afterward, the cytokine dots were visualized by ECL detection system (Millipore, Germany).

### In situ hybridization (ISH)

ISH was utilized to detect the expression of circABCC4 in tumor and adjacent tumor sections embedded in paraffin. In brief, slides were deparaffinized, rehydrated, pre-treated with 0.05% trypsin (ZSGB-BIO, ZLI-9010) at room temperature for 15 min, and fixed in 4% paraformaldehyde. Subsequently, hybridization was performed using hybridization solution (Boster, AR0152) at 52 °C, followed by hybridization. After 2 h, sections were incubated with a double-digoxigenin-labeled circABCC4 probe (QIAGEN, Germany) in hybridization solution at a final concentration of 100 nM overnight at 52 °C in a humid chamber. The sections were then washed with 2x saline-sodium citrate (SSC) buffer at 52 °C for 5 min, followed by washing with 50% deionized formamide and 2x SSC for 25 min to remove unbound probes. After blocking with 10% sheep serum (Boster, AR1009) for 1 h, sections were incubated overnight at 4 °C with anti-digoxigenin-biotin antibody (Boster, BM0040, 1:500). To visualize the labeled circular RNA, sections were first incubated with alkaline phosphatase-conjugated streptavidin (SA-AP, ZSGB-BIO, ZB-2422) at room temperature for 30 min, followed by staining with nitro blue tetrazolium/5-bromo-4-chloro-3-indolyl phosphate (NBT/BCIP, Beyotime, C3206). Sections were mounted and examined. Circ-positive expression (blue/purple staining) was detected in the cytoplasm and nucleus.

### Immunohistochemistry (IHC)

IHC was performed using paraffin-embedded samples as previously described. In brief, the specimens were incubated with antibodies specific for γH2AX, IL8 overnight at 4 °C after deparaffinization, rehydration and heat-induced antigen retrieval. Subsequently, the specimens were washed and incubated with secondary antibodies followed by DAB developer and hematoxylin. The antibodies used in this study are listed in Table S3.

### DAB Tunel Cell apoptosis detection

DAB tunel cell apoptosis detection was carried out using a DAB Tunel Cell Apoptosis Detection kit (Servicebio, Wuhan), following the manufacturer’s instructions. Briefly, after deparaffinization, rehydration, Proteinase K digestion, and blocking of endogenous peroxides, specimens were incubated with Equilibration Buffer for 10 min. Specimens were then labeled with TdT incubation buffer (Recombinant TdT enzyme: Biotin-dUTP Labeling Mix: Equilibration Buffer = 1:5:50) for 1 h at 37 °C, and subsequently stained with DAB developer and hematoxylin following Streptavidin-HRP reaction. The DAB staining resulted in the formation of a brown precipitate at the sites of DNA fragmentation, indicating apoptotic cells.

### In vivo studies

All animal studies and experimental procedures were performed under an experimental protocol approved by the Guangdong provincial people’s hospital Animal Ethics Committee. In brief, forty 4 week-old female nude mice were randomly divided into 5 groups. Orthotopic xenograft tumor models were generated with 1 $\ast$ 10^5^ lucPanc-1 cells in the same groups and treated CAFs were combined with lucPanc-1 at 1:3 ratio. 12 days after the implantation, treatment started and the first round of IVIS pictures was obtained. After 15 days of treatment, the second round of IVIS pictures was obtained, and the tumors were harvested. The xenografts were harvested, fixed in 10% formalin and embedded in paraffin for subsequent analysis. The tumor volume was measured every 3 days, and calculated with the following formular: volume (mm^3^) = (width^2^ × length)/2. At each time point of the IVIS study, 150 mg/kg D-Luciferin, potassium Salt (40902ES01, Yeasen, China) were injected i.p., and orthotopic fluorescence images were detected using an in vivo FX PRO system (BRUKER Corporation, USA). In the in vivo study, the investigators were blind to the group allocation when assessing the outcome at each time point.

### Glycolysis stress test

The Seahorse XFe96 Analyzer (Agilent) was utilized to measure the ECAR of CAFs cells following the manufacturer’s experimental protocol. In brief, CAFs cells were prepared and incubated in a CO^2^ incubator at 37 °C, while the plate was incubated in a non-CO^2^ incubator at 37 °C overnight. The next day, 10 mM glucose, 1 μM oligomycin, and 50 mM 2-DG were sequentially added to each well. The data on glycolysis, glycolytic capacity, glycolytic reserve, and non-glycolytic acidification of the cells were analyzed using the Wave software.

### Quantitative analysis of IHC and ISH

The staining scores for circABCC4, γH2AX, and IL8 were determined by considering both the staining intensity and the number of positive cells. Staining intensity was scored as follows: 0 (no staining), 1 (light), 2 (intermediate), and 3 (strong). The proportion of positively stained cells was scored as follows: 1 (<25%), 2 (25–50%), 3 (50–75%), and 4 (75–100%). The final score was calculated by multiplying the staining intensity score with the proportion of positively stained cells. The expression levels of circABCC4, IL8, and γH2AX were assessed based on the final score, using a cutoff of <4 versus ≥4.

### Statistical analysis

Five biological replicates were used in the animal study, and all other experiments adopted three biological replicates. The sample sizes were determined to ensure adequate power to detect a prespecified effect size based on the results of a preliminary investigation and experiment. All statistical analyses were performed using SPSS 12.0 software (IBM Corp, USA) and GraphPad Prism version 8.0 (GraphPad Software, USA). *T* test or one-way analysis of variance (ANOVA) was used for the comparisons of two or multiple groups, respectively. R^2^ was adopted to analyze the relationship between the IL8 IHC score and circABCC4 ISH score in the PDAC specimens. The survival curves were analyzed by using the Kaplan–Meier method with a log-rank test. All error bars represent the mean ± SD. *p* < 0.05 was considered statistically significant.

## Supplementary information

Supplemental Material

Original data (uncropped Western blots)

## Data Availability

Original images of Western Blots are provided as supplementary material. The datasets used in current study are available from the corresponding author on reasonable request.
