# Supplementary material for: Glycolysis reprogramming in CAFs promotes oxaliplatin resistance in pancreatic cancer through circABCC4 mediated PKM2 nuclear translocation
Source: Cell Death Dis. 2025 Feb 23;16(1):126. doi: 10.1038/s41419-025-07431-4 (PMC11847919; doi:10.1038/s41419-025-07431-4)
Supplement: Supplementary file 1 — Supplemental Material [file 41419_2025_7431_MOESM1_ESM.pdf]

Supplementary materials

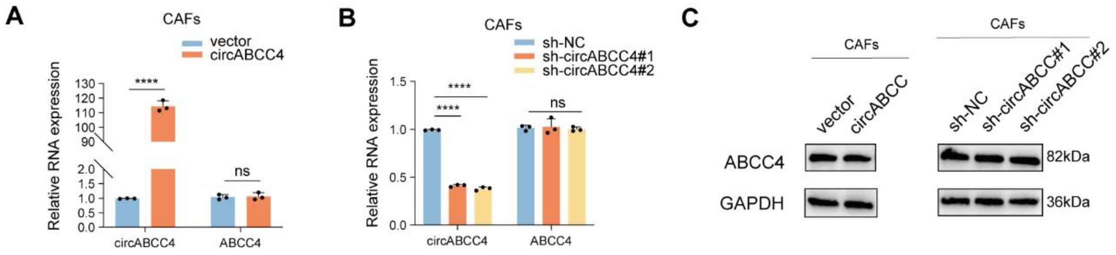

Figure S1. Specificity of circABCC4 shRNA and overexpression vector, related to Figure 2.

A-B. qRT–PCR analysis of circABCC4 and ABCC4 expression following transfecting circABCC4 shRNA and overexpression vector in CAFs.

C. Western blot analysis of ABCC4 in CAFs transfected with circABCC4 vector and in CAFs transfected with circABCC4 shRNA.

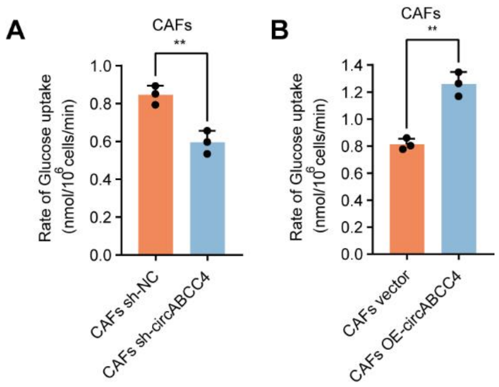

Figure S2. circABCC4 affects the glycolysis of CAFs, related to Figure 3.

A-B. The glucose uptake rate of circABCC4-silencing/overexpressing CAFs was monitored.

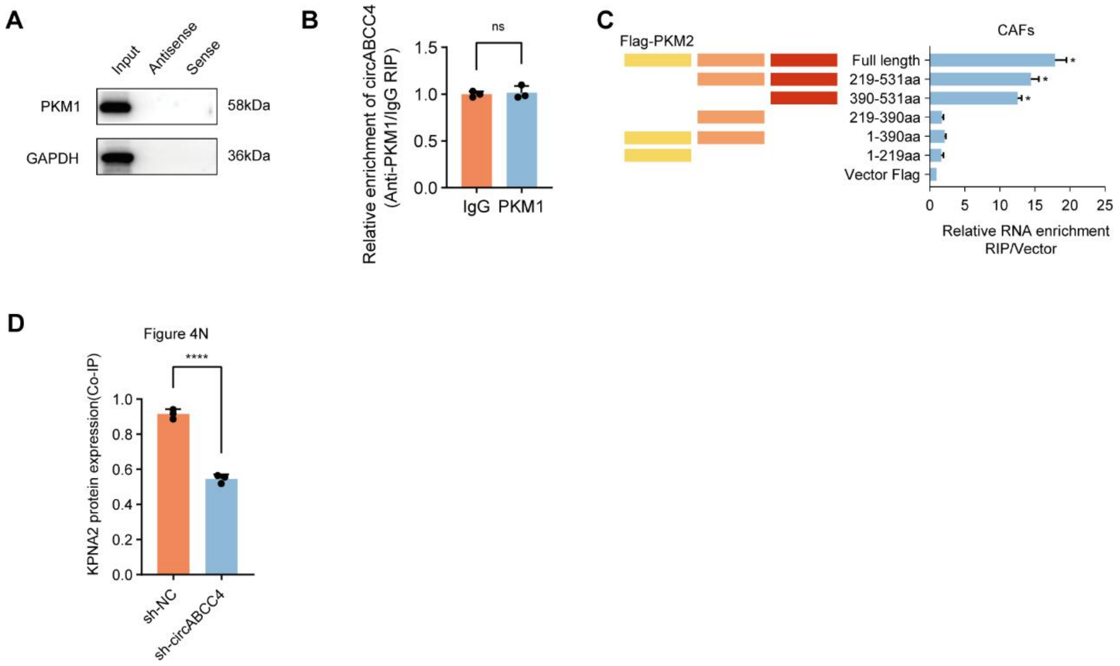

Figure S3. circABCC4 combined with PKM2 in the specific site.

A. Western blot analysis of proteins in unique differential bands. PKM1 was not identified as a candidate protein interacting with circABCC4.

B. RNA immunoprecipitation (RIP) assays in CAFs using IgG and PKM1 antibodies. The relative enrichment of circABCC4 was calculated by qRT-PCR.

C. Validation of amino acid sequences in PKM2 that bind to circABCC4. Left panel, diagrams of full-length and truncated Flag-PKM2. Right panel, qRT-PCR analysis after RIP assays of CAFs transfected with full-length and truncated Flag-PKM2.

D. Determination of the gray value of Western Blot strips related to Figure 4N.

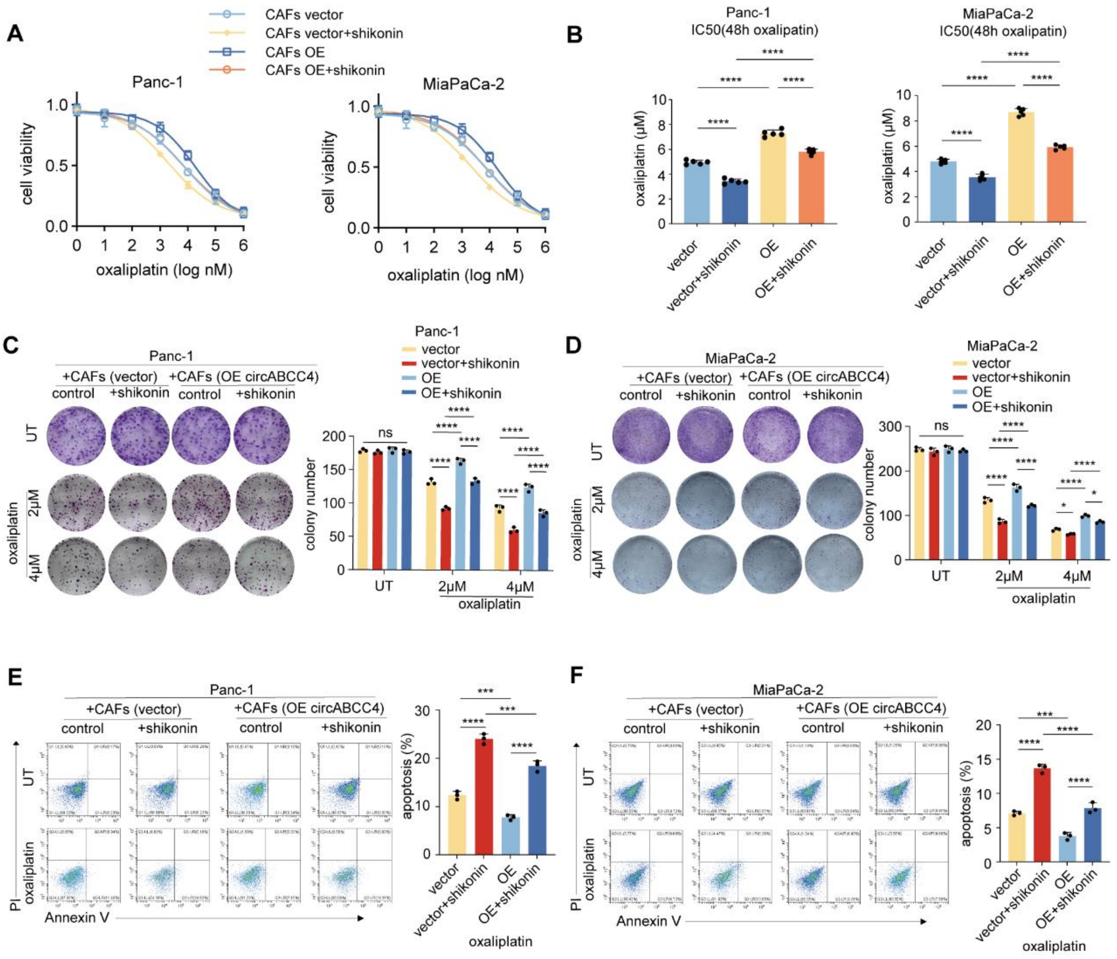

Figure S4. The inhibition of PDAC cell oxaliplatin resistance while adding shikonin in CAFs, related to Figure 4.

A-B. Oxaliplatin IC50 of Panc-1 and MiaPaCa-2 determined by constructing a dose–response curve.

C-D. Colony formation assays of Panc-1 and MiaPaCa-2 cells without or under oxaliplatin treatment (2 μM and 4 μM).

E-F. Flow cytometry analysis of oxaliplatin-induced (30μM) apoptosis in Panc-1 and MiaPaCa-2 cells. Data are expressed as the mean ± SD. *p < 0.05, **p < 0.01, ***p < 0.001, ****p <0.0001

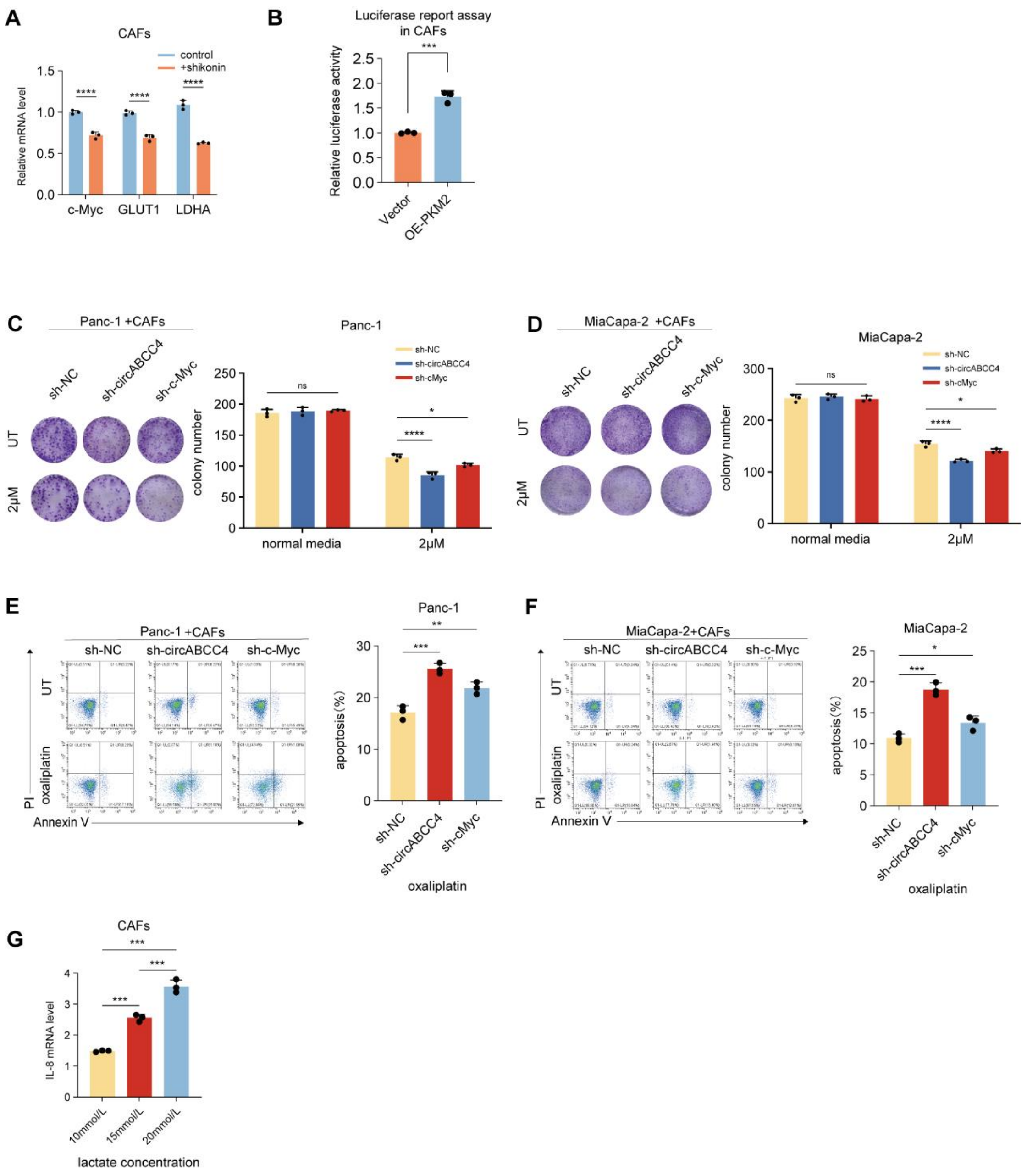

Figure S5. c-Myc is the downstream of circABCC4 and PKM2, related to Figure 5.

A. The relative mRNA levels of c-Myc, GLUT1 and LDHA were measured by qPCR in CAFs cells treated with shikonin.

B. Luciferase reporter assays of CAFs transfected with a reporter plasmid containing the c-Myc promoter, and overexpressing PKM2.

C-D. Colony formation assays of Panc-1 and MiaPaCa-2 cells under oxaliplatin treatment or without oxaliplatin.

E-F. Flow cytometry analysis of oxaliplatin-induced (30μM) apoptosis in Panc-1 and MiaPaCa-2 cells. Data are expressed as the mean ± SD. *p < 0.05, **p < 0.01, ***p < 0.001, ****p <0.0001

G. IL-8 production after adding 3 different concentrations of lactate to CAFs.

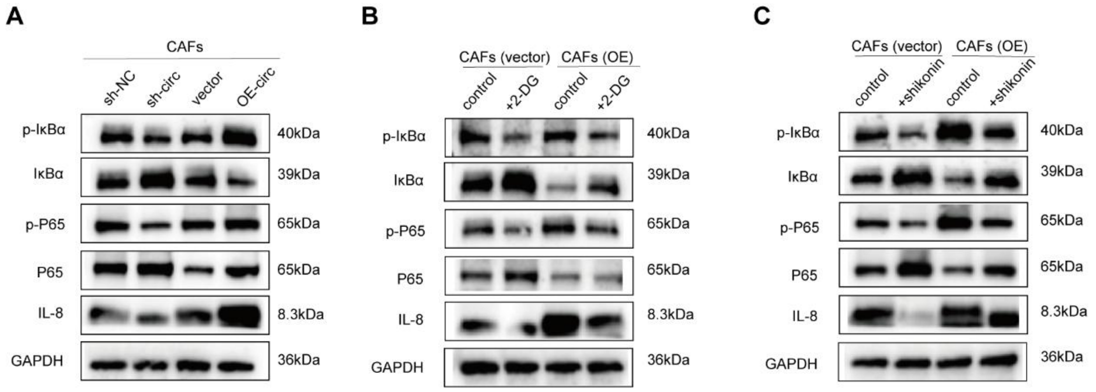

Figure S6. The activation of NF-κB pathway, related to Figure 5.

A. Western blot of the expression of p-IκBα, IκBα, p-P65, P65, IL-8 in CAFs transfected with empty vector, circABCC4, lenti-NC-shRNA, or lenti-circABCC4-shRNA.

B-C. Western blot of the expression of p-IκBα, IκBα, p-P65, P65, IL-8 in CAFs transfected with empty vector and circABCC4, as well as treated with 2-DG or shikonin.

Table S1. primer used in the experiment

Gene	Sequence (5'-3')
circABCC4-F (convergent)	AGACCCCAACTCTACAAGGC
circABCC4-R (convergent)	ATTCTTCCATGCACGCTGAC
circABCC4-F (divergent)	AAGACATTGGACACTTGGATGA
circABCC4-R (divergent)	ACAATTCGCCAGGTCTGACA
GAPDH-F (convergent)	GTCATCCCTGAGCTGAACGG
GAPDH-R (convergent)	GTCAAAGGTGGAGGAGTGGG
GAPDH-F (divergent)	CACCACACTGAATCTCCCCT
GAPDH-R (divergent)	ATTTCCTTCCCGGTTGCAAC
U6-F	CTCGCTTCGGCAGCACA
U6-R	AACGCTTCACGAATTTGCGT
ABCC4-F	GGCAGTGACGCTGTATGG
ABCC4-R	CGCCAGGTCTGACAGTAAAG
IL8-F	TAGCAAAATTGAGGCCAAGG
IL8-R	GGACTTGTGGATCCTGGCTA

Table S2. probes and oligonucleotides used in the experiments

Gene	Sequence (5'-3')	Application
Cy3-circABCC4	TTGGGG+TCTCTGA+TGCCTGGA+TGAAA+TCTA A	FISH
double-DIG-circABCC4	TCTCTGATGCCTGGATGAAATCTA	ISH
sh-circABCC4#1	AUCCAGGCAUCAGAGACCCCA	shRNA
sh-circABCC4#2	AAGACATTGGACACTTGGATGA	shRNA
circABCC4 bio-sense	CTGCCGCTGACGTTTTTAGATTTCATCCAGGCAT CAGAGACCCCAACTCTACAAGGCC	RNA-Pull down
circABCC4 bio-antisense	GGCCTTGTAGAGTTGGGGTCTCTGATGCCTGGA TGAAATCTAAAAACGTCAGCGGCAG	RNA-Pull down

Table S3 Antibody used in this study

Antibody	Application	Source
ABCC4 Rabbit Polyclonal antibody	WB, 1:1000	27167-1-AP, proteintech
Gamma H2A.X Rabbit Polyclonal antibody	WB, 1:5000<br>IF/IHC, 1:250	ab81299, abcam
Lamin B1 Rabbit Monoclonal antibody	WB, 1:1000	17416, CST
Rabbit anti-human Phospho-IκBα	WB, 1:1000	2859, CST
Rabbit anti-human IκBα	WB, 1:1000	4812, CST
Rabbit anti-human Phospho-NF-κB p65	WB, 1:1000	3033, CST
Rabbit anti-human NF-κB p65	WB, 1:1000	8242, CST
Rabbit anti-human/mouse IL-8	WB, 1:1000	27095-1-AP, proteintech

	IHC/IF, 1:250	
PKM2-Rabbit Polyclonal antibody	WB, 1:1000<br>IF, 1:250	15822-1-AP, proteintech
PKM1-Rabbit Polyclonal antibody	WB, 1:1000	15821-1-AP, proteintech
KPNA2-Rabbit Polyclonal antibody	WB, 1:1000	10819-1-AP, proteintech
Rabbit anti-human/mouse αSMA	IF, 1:2000	14395-1-AP, proteintech
GAPDH（MC4）Mouse Monoclonal Antibody	WB, 1:5000	RM2002, Beijing Ray Antibody Biotech
Goat anti-Mouse IgG(H+L)-HRP	WB, 1:5000	RM3001, Beijing Ray Antibody Biotech
Goat anti-Rabbit IgG(H+L)-HRP	WB, 1:5000	RM3002, Beijing Ray Antibody Biotech
Goat anti-rabbit Alexa Fluor 488	IF, 1:250	ab150077, abcam
