# Supplementary figures and images for: Glycolysis reprogramming in CAFs promotes oxaliplatin resistance in pancreatic cancer through circABCC4 mediated PKM2 nuclear translocation
Source: Cell Death Dis. 2025 Feb 23;16(1):126. doi: 10.1038/s41419-025-07431-4 (PMC11847919; doi:10.1038/s41419-025-07431-4)

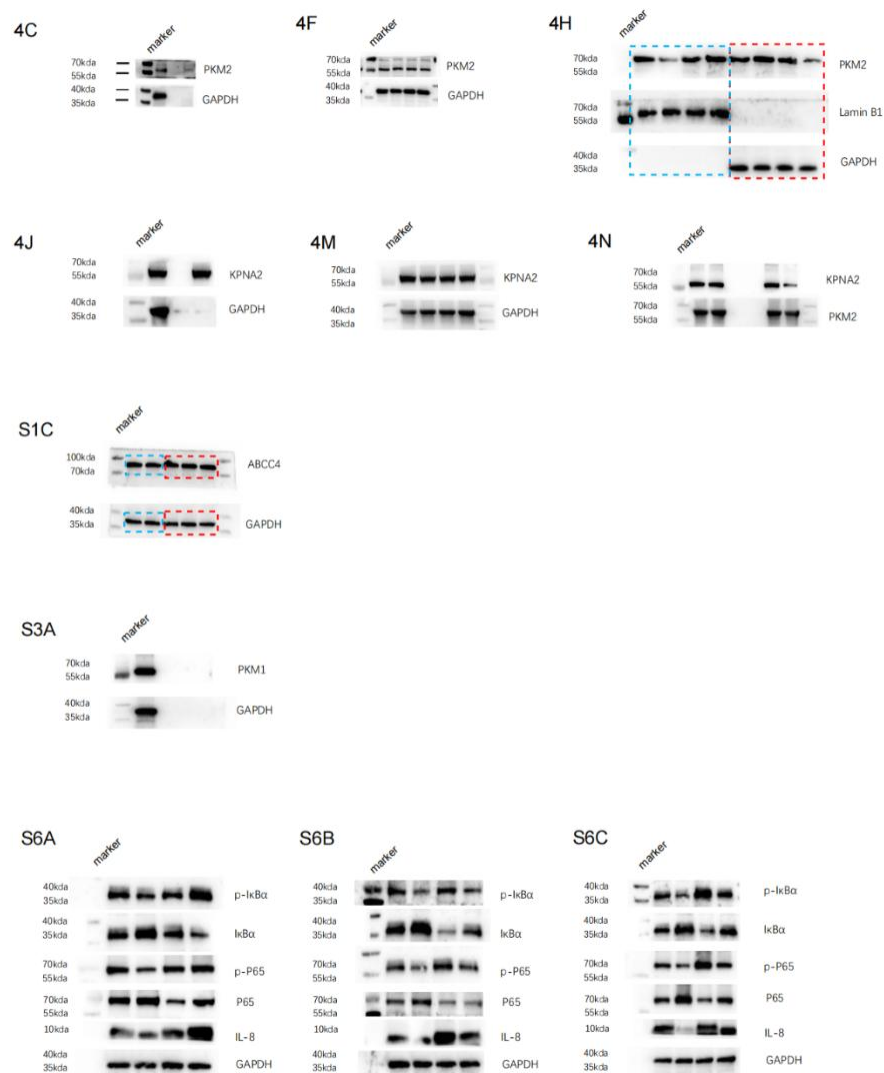

The full pictures of the blots.

Supplement: Supplementary file 2 — Original data (uncropped Western blots) [file 41419_2025_7431_MOESM2_ESM.pdf]
